# Seroepidemiology of* Hepatitis B* and* C Virus* Infections among Pregnant Women Attending Antenatal Clinic in Selected Health Facilities in East Wollega Zone, West Oromia, Ethiopia

**DOI:** 10.1155/2018/4792584

**Published:** 2018-12-10

**Authors:** Regea Dabsu, Eyasu Ejeta

**Affiliations:** ^1^Department of Medical Laboratory Sciences, Institute of Health Science, Wollega University, Nekemte, Ethiopia; ^2^Department of Medical Laboratory Sciences and Pathology, College of Health Sciences, Jimma University, Jimma, Ethiopia

## Abstract

**Background:**

Hepatitis B virus and hepatitis C virus infections are a public health problem worldwide. It is highly endemic in Asia and Sub-Saharan Africa. Horizontal and perinatal transmissions are thought to be the major modes of transmission in these countries*. Objective*. This study aimed to investigate the seroepidemiology and possible risk factors for hepatitis B virus and hepatitis C virus infection among pregnant women attending antenatal care clinics.

**Methods:**

A cross-sectional study design was conducted from July to September 2014 among 421 pregnant women attending antenatal care services in randomly selected health facilities, East Wollega Zone, West Oromia, Ethiopia. Blood sample was collected from each woman and separated serum was tested for the presence of markers. A prestructured questionnaire was used to collect sociodemographic data and risk factors. The collected data was analyzed using SPSS 20.0 statistical software. Odds ratio and 95% confidence interval were used as measures of the strength of association.

**Result:**

The overall prevalence of HBsAg was 2.4% ranging from 0.0% to 5.2%. It was the highest in Sire Health Center and the lowest/nil in others. The prevalence of HCV ranged from 6.7% to 20% with an average of 8.07% in this study area where it was highest in the Getema Health Center Antenatal Care Attendants. Only address (p=0.020) and area of the health institution (p=0.021) are significantly the associated factors for HBsAg and serostatus of HCV, respectively.

**Conclusion:**

The prevalence of HBsAg carrier rate of pregnant women in the study area falls within the medium endemic prevalence and HCV prevalence was higher than HBsAg. Study participants who were from urban areas were significantly affected with HBV while study institution affects the prevalence of HCV infection so that initiating screening tests during follow up period for antenatal care services is mandatory.

## 1. Background

Hepatitis B virus (HBV) and hepatitis C virus (HCV) infections are a major public health problem worldwide. HBV is 50 to 100 times more infectious than HIV, and it is an important cause of liver diseases such that chronic infection with HBV is a common cause of death associated with liver failure, cirrhosis, and liver cancer [1, 2]. According to the World Health Organization (WHO) 2018, an estimated 257 million people are living with hepatitis B virus infection (defined as hepatitis B surface antigen positive) [3]. The WHO also estimates that 3% of the world's populations are chronically infected with HCV; most of these cases occur in Africa, which is reported to have the highest HCV prevalence rate [4].

HBV is transmitted through parenteral or mucosal exposure to infected blood and body fluids, usually either by a vertical or horizontal route early in life in highly endemic areas, resulting in a high rate of chronic infections [5]. HBV infection during pregnancy is also associated with a high risk of maternal complications and has effects on both the mother and child [6]. The development of chronic infection is very common in infants infected from their mothers or before the age of 5 years [3].

In general, according to meta-analysis of prevalence studies of HBV infection among pregnant women, the pooled prevalence of hepatitis B virus infection among pregnant women using random-effect model was 4.7% and the overall pooled prevalence of antihepatitis C virus antibody (anti-HCV) was 3.1 % [7]. In addition to this, according to study done in Tigray regional state also the overall prevalence rate of HBV infection among the pregnant women was 5.5% in which making unprotected sexual practices with multiple partners (P = 0.03), having HBV-infected person in their family (P = 0.02), and being on history of surgical procedures (P = 0.022) were the associated risk factors [8]. The seroepidemiology of HBV infection among pregnant women is sparse and these reports were done from Jimma (3.7%, range 1.4-6.4%) and Debre-Tabor (5.3%) [9, 10].

And also the transmission of HCV from mother to child is estimated at 4–8%, but the transmission rate increases to 17–25% if the mother is also HIV infected. The worldwide prevalence of HCV infection in pregnant women is estimated to be between 1 and 8% and in children between 0.05% and 5% [11] and prevention of vertical transmission is very important, because infection at infancy usually leads to a chronic carrier status [12].

Although different studies on seroepidemiology of HBV and HCV infection in Ethiopia have been previously done in different part of the country, there is no published data on the prevalence of hepatitis B and C virus among pregnant women in East Wollega Zone administration. Thus, the current study aimed to investigate the seroepidemiology and the possible risk factors for HBV and HCV among pregnant women attending antenatal care services at selected health facilities in East Wollega Zone, West Oromia, Ethiopia.

## 2. Methods

Health facilities based cross-sectional study design was conducted in randomly selected health facilities (Nekemte Referral Hospital, Nekemte Health Center, Getema Health Center, Arjo Gudetu and Sire Health Center) in East Wollega Zone, West Oromia, Ethiopia. The zonal main city is Nekemte town which is 331km away from the capital city of Ethiopia, Addis Ababa. The town has 12 kebeles with the total population of over 94,000 and area of 58,400.000 sq. km. Nekemte Referral Hospital and Nekemte Health Center are found within the Nekemte town while Getema, Arjo Gudetu, and Sire Health Centers are located at district town level with 27Km, 42Km, and 50Km distant, respectively, from Nekemte town.

The convenient sampling method was used in which the selection of the sample was based on the easy accessibility of the pregnant women during the study period. All pregnant women attending in the randomly selected health facilities in East Wollega Zone, West Oromia, Ethiopia, during the data collection period were the source population.

The sample size was estimated with the following assumptions: an expected prevalence of 50%, margin of error of 5%, at 95% confidence level.

(1) $$\begin{array}{c} ni=\frac{Z^{2}\alpha /2P\left(1-P\right)}{d^{2}} \end{array}$$

where ni= sample population for n>10,000,* P* = prevalence of HBV/HCV antigen which is* 50*%*, d* = marginal error (0.05), and Z (*α*/2) = the reliability coefficient of 95%, i.e., 1.96.

By using this formula, the calculated sample size was 384, with the adding 10% for nonresponse, the final sample size was 422 pregnant women.

A total of 421 voluntary pregnant women attending antenatal care service in randomly selected health facilities in East Wollega Zone, West Oromia, Ethiopia, during the data collection period were the study population. The antenatal care services provider nurses were used to interview the participants and enter the data according to the prestructured questionnaire.

The sample of 5ml venous blood was collected at a spot by the investigator and was left for 30 minutes to facilitate clotting. Then the clotted blood was centrifuged to separate the serum from blood. The serum was divided into two aliquots. One of the aliquots was used for HBsAg screening and the other aliquot was used for anti-HCV antibody screening as per manufacturer instruction. The serum sample was kept in the refrigerator at 4°C and transported to Wollega University Medical Laboratory Sciences Department Laboratory. For laboratory detection of HBsAg, the SD BIOLINE HBsAg one step Hepatitis B Test is a qualitative, solid phase, two-site sandwich immunoassay for the detection of HBsAg in serum or plasma which was used as per the manufacturer protocol and Standard Operation Procedure (SOP). 

For rapid Anti-HCV test, one step HCV (Serum/plasma) 3.5mm RapiDip Insta test (one step HCV serum/plasma test strip) is a rapid immune chromatographic direct binding test for the visual detection of hepatitis C virus antibodies in serum/plasma samples in the diagnosis of hepatitis C infection and was used as per the manufacturer protocol and SOP. The test sensitivity and specificity of SD BIOLINE HBsAg one step Hepatitis B Test were 98.07% and 99.56%, respectively, while for rapid Anti-HCV test, it was 97.25 % and 99.43%, respectively. To determine the HBV/HIV and HCV/HIV coinfection, the authors used HIV test result data from the antenatal care record since the test is performed on routine basis.

Finally, each questionnaire was checked for completeness and SOP was followed during laboratory analysis. The collected data was analyzed using SPSS 20.0 statistical software. The data was organized and summarized in terms of frequencies and the results of the study were presented in a descriptive measure such as tables and graphs. The chi-square (X^2^) test and binary logistic regression (Crude odds ratio) were utilized in assessing predictor's factors of HBV and HCV infection. Any variable with a* P* value of less than 0.05 was included in the final backward multivariate logistic regression model (adjusted odd ratio). Statistically, significance was considered at* P* value less than 0.05 with 95% confidence interval (CI) level.

The study was conducted following ethical approval obtained from the Institutional Review Board (IRB) of Wollega University, Nekemet, Ethiopia. Official permission was obtained from respective study health facilities administrators and antenatal care units through an official letter of support from Wollega University Research Director. Finally, each study participant was notified about the purpose of the study, the right to refuse to participate in the study, and the anonymity and confidentiality of the information gathered.

## 3. Result

A total of 421 pregnant mothers attending ANC services in selected health institution with respondent rate of 99.76% were involved in the study. The majority of them were urban 254(60.3%), Oromo ethnic 380(90.3%), Protestant religion (59.14%), housewife or unemployed 352(83.6%), 16-24 years old 285(67.7%), married 406(96.4%), secondary and above education level 314(74.6%), first pregnant 224(53.2%), and attending the ANC services for first time 210(49.9%) (Table 1). The study participants had a mean, standard deviation, and the median age of 22.72, 3.88, and 22.0, respectively. Larger respondents had monthly average income less than 500 Ethiopian birr's, starting the ANC services after fourth and above months of pregnancy 343(81.4%). Among a total of 421 sera from pregnant women tested for HBsAg, 10 (2.4%) were positive, ranging from 0 % to 5.8% for each health institution. It was highest in Sire Health Center and nil in Nekemte Referral Hospital and Getema Health Center. At least one seropositive case for HBsAg was found in all age groups. Relatively, the highest HBV prevalence was observed from the age group of 18-24 years; however, the total number of pregnant women tested positive was only six, from 285 tested pregnant women among this specified age group. Relatively, the highest HBV prevalence was observed from the age group of 18-24 years.

There was no statistically significant difference between the prevalence of HBsAg and age in all the study sites. The seroprevalence of HBsAg in relation to age is shown in Table 1. The sociodemographic status of the study population shows that a high proportion of HBsAg positivity was among the illiterate 3/107(2.8%), those whose income was 501-1,499 Birr/month 4/150(2.7%), and other ethnic subgroups 1/19(5.3%). However, the overall prevalence of HBsAg among the illiterate (2.8%) and other ethnic groups was 5.3% and has no significant difference (p> 0.05) when compared with other educational status and ethnic groups, respectively (Table 1). Only living in urban areas is a potential risk of HBV infection, to the population evaluated (P=0.020). It has not statistically significant association with any of the potential risk factors for HBV infections among study participants in the study area (Table 2).

The prevalence of HCV ranged from 6.7% to 20% with an average of 8.07% in this study area where it was highest prevailing in the Getema Health Center and lower in Nekemte Referral Hospital ANC attendants. The HCV serostatus is higher among rural resident (10.2%), employed (13.0%), monthly income less than 500 attending higher education level. But the HCV serostatus was only statistically significant associated with area of study institution from the sociodemography of the study participant in bivariate analysis (P=0.021) (Table 1). It has not a statistically significant association with any of the potential risk factors for HCV even though it was higher among participants performing Ear/Nose piercing in jeweler's shop (11.1%), having dental extraction at health facility (8.8%), being circumcised (8.1%), shaving eyebrow (15.6%), receiving blood transfusion (12.5%), having multiple sexual partners (11.1%), and having history of STD (venereal disease) (10.4%) (Table 3). In the multivariate logistic analysis, the HCV serostatus is associated with the only area of health institution (adjust odd ratio at 95 confident interval= 204(.053, .788)) (Table 1). In general, the overall coinfection rate of HBV/HCV among tested pregnant women is 0.24 % (1/421) whereas the overall HCV-HIV coinfection is 2.9% while the overall HIV prevalence among the studied participant is 0.24%.

## 4. Discussion

This study investigated the seroepidemiology of HBV and HCV infection among pregnant women in the study area. The overall prevalence of HBsAg and anti-HCV in pregnant women from the present study site was 2.4% and 8.07%, respectively. This is comparable with a study done previously in Ethiopia in which the prevalence of hepatitis B virus infection among pregnant women was (4.7%) [13]. But the prevalence of HCV was higher than the previous study (3.1%) [14]. In the present study, the overall prevalence of HBV infections was 2.4% which was lower than that of the study done in Dessie Referral Hospital, Ethiopia, in which the overall prevalence of HBV infections was 4.9 % [10]. This might be due to the difference in the health facility and study area. But this is in contrast with the previous study done elsewhere in which 12.5% of pregnant women were found to be positive for HBsAg [15]. This is also in contrast with study done in different part of Ethiopia that revealed the prevalence of HBV and HCV in the overall pooled prevalence of hepatitis B virus (HBV) was 7.4% and the overall pooled prevalence of antihepatitis C virus antibody (anti-HCV) was 3.1% [7, 14] and also with study done in Nigeria with seroepidemiology of HBV and HCV as 6.78% and 1.39%, respectively [16].

In the present study, the prevalence HBV of each study area differs from one another. It was highest relatively in Sire Health Center and almost nil in Getema Health Center and Nekemte Referral Hospital (Sire Health Center=5.2%, Arjo Gudetu Health Center=3.8%, Nekemte Health center=2.0%). This is comparable with the previous study done in Felege Hiwot Referral Hospital, northwest Ethiopia, in which the overall prevalence of HBsAg was 4.4% [17] and also in Eriteria the prevalence of HBV showed marked difference among the regions ranging from 2.1% to 7.5%. [18]. But the prevalence was lower when compared with a study done in Nigeria and Uganda in which the prevalence was 4.7% and 6.3% [16, 19]. This might be due to the small sample size in the present study. The overall coinfection prevalence of HBV/HIV was 4 (1%) which was lower than the previous study done in different parts of Ethiopia and Rwanda in which the prevalence is 2.1%, 22.2 %, and 4.1% [20, 21]. This difference might be due to the level of awareness and behavioral characteristics of the study participant.

The prevalence of HBV infections was not significant among patients who had a history of multiple sexual practices, nose piercing, and no formal education. This is similar to meta-analysis study done in other parts of Ethiopia and elsewhere in which among study parameters, only study years were associated with a decreasing HBV prevalence rate over time [7, 15] and also in line with a study done in southern Ethiopia in which neither the type of risk factors nor exposure to multiple risk factors was significantly associated with HBV infection [22] but inconsistent with the previous study done in Addis Ababa in which history of abortion and history of surgery and tattooing were significantly associated with HBV infection [21].

In the present study, relatively, the highest HBV prevalence was observed from the age group of 18-24 years, but there was no statistically significant difference between the prevalence of HBsAg and age in all the study sites. This is in line with the previous study in which prevalence of HBV did not vary significantly by age. Similarly, high prevalence of HBV infection was investigated among pregnant women who were from an urban setting which is similar with previous study done in Ethiopia [21].

In this study, sociodemographic and obstetrical characteristics of pregnant women assessed were not associated with HBsAg positivity which is similar to a study done in Yirgalem Hospital, Ethiopia [20]. This is also in line with a study done in Iran in which there was no significant association between HBsAg results with age, mother's educational level, place of residence, history of cesarean section, and blood transfusion [23]. But this is in contrast with a study done in Addis Ababa in which seropositivity for hepatitis B surface antigen was statistically associated with a history of abortion surgery and family history for hepatitis [21]. It is also in contrast with the previous study done in Dessie Referral Hospital [10] and Harar City, Ethiopia, in which HIV positive previous history, history of blood transfusion, history of surgical procedure, history of STI infection, previous history of tooth extraction, and history of multiple sexual partners were significant for acquiring HBV infection [14].

The overall prevalence of HCV among pregnant women attending ANC services in the study institution was 8.07% differing among the health institution with high prevalence among Getema Health Center which is inconsistent with the study done in rural communities of Abaji Area Council, Nigeria [16]. This observed difference could be related to our study area that includes both rural and urban communities. In this study, there was a significant difference between the study institutions where seroepidemiology ranges from 20.7% to 5.1%. The variation might be due to geographical situation, the level of awareness, cultural and behavioral differences, and the difference in hepatitis epidemiology, tribal practices, traditional operation, sexual practices, and medical exposure for the potential risk factors of HCV infection. This will call for future research to identify the cause of the significant difference and other related factors in the study area. And also a community-based study of seroepidemiology of both viruses is also important for further intervention. The overall prevalence of HCV infections is greater than a study done in Dessie Referral Hospital (0.8%) and Bahir Dar health institutions (0.6%), Ethiopia [10, 24]. In the present study on prevalence of HCV, none of the expected risk factors have been found to be associated with HCV positivity which is similar to a study done in Bahir Dar health institutions [24].

In the present study result, HBV/HCV coinfection among HBV positive pregnant women evaluated was 10% (1/9). This is higher than previous study done elsewhere in which 0.57% was found to have mixed infections of hepatitis B and C viruses [15]. The overall coinfection rate of HBV/HCV among tested pregnant women was 0.24 % (1/421). This is comparable with a study done in Nigeria in which no woman was coinfected with the two viruses [25].

Regarding HBV/HIV coinfection in the present study, the prevalence of coinfection was 4 (1%) which is in contrast with a previous study done in southern Ethiopia in which HBV/HIV coinfection was 14.2% [22]. This variation might be due to the difference in hepatitis epidemiology, sexual practices, and medical exposure for the potential risk factors which may need further study.

The overall HCV-HIV coinfection is 2.9% while the overall HIV prevalence among the studied participant was 0.24%. This is in agreement with study done in other parts of Ethiopia in which all HCV positivity was high among pregnant women coinfected with HIV [7, 10] and also similar with study done in Rwanda (3.9%), but lower than study done in Kenya in which the coinfection of HIV and HCV was 10.3% [26].

In the multivariate logistic analysis, the HCV serostatus is associated with the only area of health institution (adjust odd ratio at 95 confident interval= 204(.053, .788)) which is in agreement with previous study done elsewhere in which the risk factors variables did not have significant association with HCV positive status [27] in contrast with a previous study in which women in urban residence were more likely to be associated with HCV infection compared to those living in rural setting [28].

This study finding tells that there shall be a screening test for all pregnant women attending antenatal care clinics to get care services to prevent the complication of these viruses on pregnant women as well as on fetus. Most of the suspected risk factors for both hepatitis B viruses and hepatitis C virus transmission and infection for the study participants remained unrelated to be possible factors and needed further investigation. This is in line with a study done in Nigeria [25].

The strength of this study is that the response rate was 99.8% and different study sites were included in the study. As weakness in this study, correlation of clinical manifestation and laboratory investigation is not made. Since the study design was cross-sectional, it shares the shortcoming of constructing cause and effect relationship. And also as a seroepidemiology, health institution based result has less weight as compared with a community-based study. Because of lack of facilities, HBeAg test was not performed and the birth outcome was not evaluated. And also viremia status of patients was not conducted due to lack of facilities.

## 5. Conclusion

The prevalence of HBsAg carrier rate of pregnant women in the study area falls within the medium/intermediate endemic according to the criteria set by WHO, but the prevalence HCV level was also higher than HBsAg. Study participants who were from urban areas were significantly affected with HBV while study institution affects the prevalence of HCV infection. Therefore, initiating screening tests during follow up period for antenatal care services is mandatory. And further study with a large scale sample size on HBsAg and HCV positivity is required to enrich the data in the study area.

## Figures and Tables

**Table 1:** Prevalence of HBs Ag and HCV infection in relation to sociodemographic characteristics of pregnant women attending the selected health facilities in East Wollega, West Oromia, Ethiopia, from July to September, 2014.

Characteristics		Total, n (%)	HBV infection			HCV infection		
			Positive, n (%)	COR (95%CI)	P-value	Positive, n (%)	COR (95%CI)	P-value
Address	Urban	254(60.3)	2(0.8)	**0.16(0.03-0.75)** **∗**	**0.020**	17(6.7)	0.63(0.31-1.27)	0.202
	Rural	167(39.7)	8(4.8)	1		17(10.2)	1	
Occupation	Employed	69(16.4)	1(1.4)	0.56(0.07-4.49)	0.586	9(13.0)	1.96(0.87-4.41)	0.103
	House wife	352(83.6)	9(2.6)	1		25(7.1)	1	
Monthly Income	≤500	207(49.2)	5(2.4)	0.88(0.36-2.15)	0.688	21(10.1)	1.69(0.56-5.13)	0.351
	501-1, 499	150(35.6)	4(2.7)	0.58(0.06-5.28)	0.629	9(6.0)	0.95(0.28-3.23)	0.944
	≥1, 500	64(15.2)	1(1.6)	1		4(6.2)	1	
Maternal age	18-24	285(67.7)	6(2.1)	1.55(0.18-13.59)	0.281	23(8.1)	1.22(0.15-9.77)	0.845
	25-31	121(28.7)	3(2.5)	1.73(0.18-15.75)	0.385	10(8.3)	1.26(0.15-10.61)	0.831
	≥31	15(3.6)	1(6.7)	1		1(6.7)	1	
Education level	Illiterate	107(25.4)	3(2.8)	1.15(0.25-5.23)	0.860	5(4.7)	0.35(0.12-1.09)	0.072
	Primary	151(35.9)	3(2.0)	0.81(0.17-3.66)	0.780	13(8.6)	0.94(0.44-1.99)	0.870
	2° and above	163(38.7)	4(2.5)	1		16(9.8)		
Religion	Orthodox	120(28.5)	4(3.3)	1.40(0.39-5.06)	0.606	10(8.3)	1.10(0.49-2.45)	0.806
	Muslim	46(10.9)	0(0.0)	0(0)		4(8.7)	1.16(0.37-3.57)	0.799
	Other	5(1.2)	0(0.0)	0(0)		1(20.0)	3.04(0.32-28.57)	0.331
	Protestant	250(59.4)	6(2.4)	1		19(7.6)	1	
Ethnic	Amhara	22(5.2)	1(4.5)	2.21(0.26-18.54)	0.463	1(4.5)	0.54(0.07-4.12)	0.549
	Other	19(4.5)	1(5.3)	2.58(0.31-21.78)	0.383	2(10.5)	1.32(0.29-5.99)	0.715
	Oromo	380(90.3)	8(2.1)	1		31(8.2)	1	
Marital status	Married	406(96.4)	9 (2.2)	0.312(0.04-2.68)	0.292	32(7.9)	0.55(0.12-2.57)	0.453
	Other*∗∗*	15(3.6)	1(6.7)	1		2(13.3)	1	
Gestational stage	1st trimester	66(15.7)	2(3.0)	1.74(0.28-10.65)	0.549	7(10.6)	1.14(0.45-2.92)	0.781
	2nd trimester	185(43.9)	5(2.7)	1.55(0.36-6.57)	0.555	11(5.9)	0.61(0.27-1.35)	0.222
	3rd trimester	170(40.4)	3(1.8)	1		16(9.4)	1	
Parity	Primigravida	220(52.3)	4(1.8)	0.59(0.11-3.31)	0.551	8(10.3)	2.10(0.60-7.30)	0.243
	Gravidity	135(32.1)	4(3.0)	0.97(0.17-5.47)	0.979	15(7.9)	1.86(0.50-6.92)	0.353
	Multigravid	66(15.7)	2(3.0)	1		11(7.2)	1	
Number of live birth	(0)1st pregnancy	224(53.2)	4(1.8)	0.45(0.08-2.55)	0.370	20(9.1)	2.58(0.58-11.39)	0.209
	≤2	145(34.4)	4(2.8)	0.71(0.13-.99)	0.697	11(8.1)	2.052(0.44-9.58)	0.361
	≥3	52(12.4)	2(3.8)	1		3(4.5)	1	
Study Institutions	NHC	150(35.7)	3(2.0)	0.52(0.10-2.62)	0.426	10(6.7)	1.34(0.41-4.41)	0.631
	NRH	85(20.2)	0(0.0)	0(0)	0.997	8(9.3)	1.92(0.55-6.65)	0.302
	GHC	29(6.9)	0(0.0)	0(0)	0.998	6(20.7)	**4.89(1.27-18.84)** **∗**	**0.021**
	SHC	77(18.3)	4(5.2)	1.38(0.30-6.42)	0.675	6(7.8)	1.58(0.43-5.85)	0.490
	AGHC	79(18.8)	3(3.8)	1		4(5.1)	1	

*∗=statically significant (P<0.05), 1 =reference group, COR= crude odd ratio, AOR=adjusted odd ratio 95*%, *C.I=95*%* confidence interval*, *NHC*=Nekemte Health Center, *NRH*= Nekemte Referral Hospital, *GHC*=Getema Health Center, *SHC*=Sire Health Center, *AGHC*=Arjio Gudatu Health Center.

**Table 2:** Potential risk factors and prevalence of HBVsAg among pregnant women attending selected health facilities in East Wollega, West Oromia, Ethiopia, from July to September, 2014.

Risk factors	Response	HBsAg positive, n (%)		Total, n (%)	X^2^	p-value
		Positive	Negative			
Ear/Nose piercing in jeweler's shop	Yes	0(0.0)	8 (100.0)	8((1.9)	0.198	0.656
	No	10(2.4)	403(95.7)	413(98.1)		
Tattooing on body	Yes	5(3.2)	149(96.8)	154(36.6)	0.795	0.373
	No	5(1.9)	262(98.1)	267(63.4)		
Dental extraction at home	Yes	2(3.6)	53(96.4)	55(13.1)	0.455	0.797
	No	8(2.2)	357(97.8)	365(86.7)		
Dental extraction at health facility	Yes	00(0.0)	102(100.0)	102(24.2)	3.275	0.070
	No	10(3.1)	309(96.9)	319(75.8)		
Circumcised	Yes	9(2.4)	361(97.6)	370(87.9)	0.043	0.836
	No	1(2.0)	50(98.0)	51(12.1)		
Abortion	Yes	0(0.0)	35(100.0)	35(8.3)	0.929	0.335
	No	10(2.6)	376(97.4)	386(91.7)		
History of STD	Yes	0(0.0)	48(100.0)	48(11.4)	1.31	0.251
	No	10(2.7)	363(86.2)	373(88.6)		
Shaving eye brow	Yes	00(0.0)	32(100.0)	32(7.6)	0.843	0.359
	No	10(2.6)	379(97.4)	389(92.4)		
Delivery by TBA (trained birth attendant)	Yes	4(5.2)	73(94.8)	77(18.3)	3.23	0.072
	No	6(1.7)	338(98.3)	344(81.7)		
Pervious delivery at health facility	Yes	2(2.5)	78(97.5)	80(19.0)	1.144	0.565
	No	8(2.7)	291(97.3)	299(71.0)		
Hospital admission	Yes	0(0.0)	46(100.0)	46(10.9)	1.257	0.262
	No	10(2.7)	365(97.3)	375(89.1)		
Surgical procedure	Yes	0(0.0)	18(100.0)	18(4.3)	0.458	0.0499
	No	10(2.5)	393(97.5)	403(95.7)		
Receiving blood transfusion	Yes	0(0.0)	8(100.0)	8(1.9)	0.198	0.656
	No	10(2.4)	403(97.6)	413(98.1)		
History of Contact with jaundiced Patient/Liver disease	Yes	1(4.3)	22(95.7)	23(5.5)	0.408	0.523
	No	9(2.3)	389(97.7)	398(94.5)		
Home delivery	Yes	4(3.9)	99(96.1)	103(24.5)	1.338	0.247
	No	6(1.9)	312(98.1)	318(75.5)		
Previous Caesarian section	Yes	0(0.0)	38(100.0)	38(9.0)	1.016	0.313
	No	10(2.6)	373(97.4)	383(91.0)		
Venous or body piercing for treatment	Yes	0(0.0)	59(100.0)	59(14.0)	0.015	0.904
	No	10(2.8)	352(83.6)	362(86.0)		
Multiple Sexual partners	Yes	0(0.0)	9(100.0)	9(2.1)	0.224	0.636
	No	10(2.4)	402(97.6)	412(97.9)		

**Table 3:** Host related associated factors for prevalence of HCV among pregnant women attending ANC services at selected health facilities, East Wollega, West Oromia, Ethiopia, from July to September, 2014.

Risk factors	Response	HCV sero-status. n(%)		X^2^	p-value
		Positive	Negative		
Ear/ Nose piercing (in jeweler's shop)	Yes	1(11.1)	8(88.9)	0.114	0.736
	No	33(8.0)	379(92.0)		
Tattooing on body	Yes	14(9.1)	140(90.9)	0.337	0.562
	No	20(7.5)	247(92.5)		
Dental extraction at health facility	Yes	6(5.9)	96(94.1)	0.873	0.350
	No	28(8.8)	291(91.2)		
Circumcised	Yes	30(8.1)	340(91.9)	0.004	0.948
	No	4(7.8)	47(92.2)		
Abortion	Yes	2(5.7)	33(94.3)	0.287	0.592
	No	32(8.3)	354(91.7)		
Shaving eye brow	Yes	5(15.6)	27(84.4)	2.658	0.103
	No	29(7.5)	360(92.5)		
Pervious delivery at health facility	Yes	5(6.2)	75(93.8)	0.563	0.755
	No	26(8.7)	273(91.3)		
Hospital admission	Yes	2(4.3)	44(95.7)	0.967	0.325
	No	32(8.5)	343(91.5)		
Surgical procedure	Yes	1(5.6)	17(94.4)	0.161	0.688
	No	33(8.2)	370(91.8)		
Receiving blood transfusion	Yes	1(12.5)	7(87.5)	0.215	0.643
	No	33(8.0)	380(92.0)		
History of Contact with jaundiced Patient	Yes	1(4.3)	22(95.7)	0.456	0.500
	No	33(8.3)	365(91.7)		
Previous Caesarian section	Yes	1(2.6)	37(97.4)	1.668	0.197
	No	33(8.6)	350(91.4)		
Venous or body piercing for treatment	Yes	5(8.5)	54(91.5)	0.015	0.904
	No	29(8.0)	333(92.0)		
Multiple Sexual partners	Yes	1(11.1)	8(88.9)	0.114	0.736
	No	33(8.0)	379(92.0)		
History of STD (Venereal disease)	Yes	5(10.4)	43(89.6)	.400	.527
	No	29(7.8)	344(92.2)		

STD: sexually transmitted disease, ANC: ante natal care.

## Data Availability

The survey questioners data used to support the findings of this study are included within the supplementary information file.

## Supplementary Materials

Supplementary Materials Details of tools or questionnaires used in this research work for gathering information/data on sociodemographic background, possible risk factors, and laboratory investigation report for each study participant are provided.
