# Supplementary material for: Seroepidemiology of Hepatitis B and C Virus Infections among Pregnant Women Attending Antenatal Clinic in Selected Health Facilities in East Wollega Zone, West Oromia, Ethiopia
Source: Biomed Res Int. 2018 Dec 10;2018:4792584. doi: 10.1155/2018/4792584 (PMC6311240; doi:10.1155/2018/4792584)
Supplement: Supplementary Materials — Details of tools or questionnaires used in this research work for gathering information/data on sociodemographic background, possible risk factors, and laboratory investigation report for each study participant are provided. [file 4792584.f1.docx]

The following document is the details of tools or questionnaires used in this research work for gathering information/data on socio-demographic background, possible risk factors and laboratory investigation report for each study participant(***Supplementary file for this work***).

Organization:- Wollega University, Institute of Health Science, Department of Medical Laboratory Science

Title of the study:- Sero-Epidemiology of *Hepatitis B* and *C Virus* Infections among Pregnant Women Attending Antenatal Clinic in Selected Health Facilities in East Wollega Zone, West Oromia, Ethiopia which is the major cause of morbidity and morbidity, and for provision of recommendation for possible prevention and controlling the problem and help them for effective intervention plan in the future.

The involvement in this study is based on your voluntary and you have the right to refuse to participate in the study, and the confidentiality of the information gathered will be kept and only used for this study. The result of the laboratory finding will be communicated to your physician or care giver.

**Direction:-**Please encircle the letter of your answer or correctly fill in the blank space provided for open ended questioners.

## Part-I: Socio demography survey questioner

1. Identification: Date _________________ Code no ______________

Address________________Age __________________

2. Where do you live? A. Urban B. Rural

3 Highest educational attainments

1. Illiterate B. Primary C. Secondary

D-Higher education E.Other, specify _____________________

4. What is your occupation?

1. Farmer B. Housewife C. Private
2. Daily laborer E. Governmental EmployeeF. NGO employee

F-Other, specify ____________________

5. Religion

1. Orthodox B. Moslem C. Protestant

D-Catholic Other, specify ____________________

6. Ethnicity

1. Amhara B. Tigray C. Oromo D. Other, specify ____________

7. What is your marital status?

1. Married B. Unmarried

8. Family Monthly income (specify) ________________________

9. Family numbers________________________

10. Number of children alive________________________

11. Parity A. primigravidae B. Multigravidae, C. Grand multipara

**Gestation at first ANC visit _________________________**

Number of ANC visits on the current pregnancy

1. One B. Two up to three C. Four and greater

12. Your gestational stage (trimesters)? A. First B. Second C. Third

**Part II: Questions related to HBV and HCV risk factors**

13. Have you have or ever practiced the following?

14. Ear/Nose piercing (in jeweler’s shop) A. yes B. No

16. Tattooing on body A. yes B. No

17. Dental extraction at home A. yes B. No

18. Dental extraction at health facility A. yes B. No

19. Circumcision A. yes B. No

20. Shaving A. yes B. No

21. Delivery by TBA (trained birth attendant) A. yes B. No

22. Pervious delivery at health facility A. yes B. No

23. Abortion A. yes B. No

24. Hospital admission A. yes B. No If yes why?_________

24. Surgical procedure A. yes B. No

25. Receiving blood transfusion A. yes B. No

26. History of Contact with jaundiced A. yes B. No

Patient/Liver disease

27. Home delivery A. yes B. No

28. PreviousCaesarian section A. yes B. No

29. Using Loop contrastive A. yes B. No

30. Injectable contrastive A. yes B. No

31. Venous or body piercing for treatment A. yes B. No

33. Multiple Sexual partners A. yes B. No

34. History of STD (Venereal disease) A. yes B. No

35. No risk A. yes B. No

**36. Laboratory result**

| Test type | HIV test result | | HBVsAg test result | | Anti-HCV test result | |
| --- | --- | --- | --- | --- | --- | --- |
| Laboratory test result | +Ve | -Ve | +Ve | -Ve | +Ve | -Ve |
|  |  |  |  |  |  |  |

We thank you for your response!
